# A New Approach to Inhibiting Triple-Negative Breast Cancer: In Vitro, Ex Vivo and In Vivo Antiangiogenic Effect of BthTx-II, a PLA_2_-Asp-49 from *Bothrops jararacussu* Venom

**DOI:** 10.3390/biom12020258

**Published:** 2022-02-04

**Authors:** Fernanda Van Petten de Vasconcelos Azevedo, Daiana Silva Lopes, Mariana Alves Pereira Zóia, Lucas Ian Veloso Correia, Natieli Saito, Belchiolina Beatriz Fonseca, Lorena Polloni, Samuel Cota Teixeira, Luiz Ricardo Goulart, Veridiana de Melo Rodrigues Ávila

**Affiliations:** 1 Laboratory of Biochemistry and Animal Toxins, Institute of Biotechnology, Federal University of Uberlandia, Uberlândia 38408-100, MG, Brazil; lucasian.veloso@gmail.com (L.I.V.C.); polloni.lorena@gmail.com (L.P.); 2 Laboratory of Nanobiotechnology, Institute of Biotechnology, Federal University of Uberlandia, Uberlândia 38408-100, MG, Brazil; marianazoia@hotmail.com (M.A.P.Z.); natielisaito@gmail.com (N.S.); lrgoulart@ufu.br (L.R.G.); 3 Multidisciplinary Institute of Health, Federal University of Bahia, Vitoria da Conquista, Salvador 40170-110, BA, Brazil; lsdaiana@yahoo.com.br; 4 College of Veterinary Medicine, Federal University of Uberlandia, Uberlândia 38408-100, MG, Brazil; biafonseca@ufu.br; 5 Department of Immunology, Biomedical Sciences Institute, Federal University of Uberlandia, Uberlândia 38408-100, MG, Brazil; samuctx@gmail.com

**Keywords:** Asp-49 PLA_2_, BthTx-II, breast cancer, angiogenesis, phospholipase A_2_

## Abstract

Phospholipases A_2_ (PLA_2_) represent a superfamily of enzymes widely distributed in living organisms, with a broad spectrum of pharmacological activities and therapeutic potential. Anti-angiogenic strategies have become one of the main tools in fighting cancer. In this sense, the present work reports the inhibition of tumor angiogenesis induced by Asp-49 BthTX-II using in vitro, ex vivo and in vivo approaches. We demonstrate that BthTx-II inhibited cell adhesion, proliferation, and migration of human umbilical vein endothelial cells (HUVEC), as well as caused a reduction in the levels of endothelial growth factor (VEGF) during in vitro angiogenesis assays. BthTx-II was also able to inhibit the sprouting angiogenic process, by the ex vivo germination assay of the aortic ring; in addition, this toxin inhibited the migration and proliferation of HUVEC in co-culture with triple-negative breast cancer cells (e.g., MDA-MB-231 cells). Finally, in vivo tumor suppression and anti-angiogenic activities were analyzed using MDA-MB-231 cells with Matrigel injected into the chorioallantoic membrane of chicken embryo (CAM) for 7 days treatment with BthTx-II, showing a considerable reduction in vessel caliber, on the size and weight of tumors. Together, these results suggest an important antiangiogenic and antitumor role for BthTx-II, as a potential prototype for the development of new tools and antitumor drugs in cancer therapy.

## 1. Introduction

Breast cancer is the leading cause of cancer death among women worldwide and new cases are increasing each year, according to the National Center for Health Statistics [1]. It is considered a heterogeneous disease with different distinct subtypes that respond differently to pharmacological therapy with variable rates of failure and mortality, and this issue may be associated with the capacity of formation of new vessels and tumor metastasis [2].

Physiological angiogenesis is known to occur mainly during embryogenesis whose function is to accommodate developmental requirements and, for this reason, only 0.01% of epithelial cells (ECs) undergo cell division in adults [3]. However, angiogenesis plays a critical role in a variety of pathological conditions, and we have already reported on how important this new network for nutrition is in cancer. However, when this supply is insufficient, it can lead to apoptosis or necrosis of tumor cells, depending on the aggressiveness of the carcinogens and the environment in which it is inserted [4]. Therefore, the development of new agents capable of inhibiting this angiogenesis and reducing the aggressiveness of cancer represents a new paradigm in cancer prevention and treatment.

The process of tumor growth is determined by the physical dissemination of the cell from its place of origin, with the ability to grow, multiply and invade distant sites. Thus, to invade the local parenchyma and circulatory system, and to eventually reach the secondary site, tumor cells need to have intrinsic metastatic properties. In this scenario, tumor angiogenesis triggers an important function in the tumorigenic process. Tumors progress from an avascular to a vascular state in response to micro environmental changes, specifically the onset of severe hypoxia [5,6].

Tumor angiogenesis is a complex process characterized by a sequence of interactions between endothelial cells and tumor cells, resulting in the formation of vessels adjacent to the primary tumor. The presence of new vessels in this region facilitates both tumor growth and metastasis, which taken together are considered one of the hallmarks of cancer, especially in solid tumors, accounting for 90% of deaths related to triple-negative breast cancer (TNBC) [7]. This profile is closely related to the tumor microenvironment, composed of fibroblasts, inflammatory cells, perivascular cells, and endothelial cells. Therefore, many studies in the current literature have focused on the inhibition of tumor angiogenesis as a strategy for the treatment of this type of tumor [5,8].

The most of conventional models for studying in vitro angiogenesis are based on the use of endothelial cells in compliant matrices, such as collagen, fibrin or Matrigel, which are related to known pro-angiogenic effects. Although these models are able to provide insights into the angiogenesis process, there is still a lack of understanding of the underlying mechanisms related to the direct interaction between endothelial cells and tumor cells. In this context, the development of three-dimensional in vitro (3D) tumor models that reproduce the physiological responses of the tumor microenvironment in vivo has emerged as an interesting tool for better understanding angiogenesis, as well as serving as a suitable approach for testing new antiangiogenic drugs [9,10,11,12,13].

Over the decades, literature findings have demonstrated snake venoms as a potential resource for the design of new drugs. Among the complex mixture of biologically active molecules present in snake venoms, the snake venom phospholipase A_2_ (svPLA_2_) is considered one of the most studied toxins. Previous studies have shown that purified svPLA_2_s possess antitumor and antiangiogenic properties [14,15,16,17,18]. BnSP-7, a Lys49-PLA_2_ isolated from *Bothrops pauloensis* snake venom inhibited the adhesion and migration of HUVEC, and impaired in vitro and ex vivo angiogenesis in a VEGF-dependent manner [14]. CC-PLA_2_-1 and CC-PLA_2_-2, two secreted PLA_2_s purified from *Cerastes cerastes* venom waxes, demonstrated dose-dependent inhibition of human brain microvascular endothelial cell adhesion and migration [19]. MVL-PLA_2_, an acid Asp49 PLA_2_ from *Macrovipera lebetina* venom, exhibited anti-integrin activity by inhibiting adhesion and migration of human tumor and microvascular endothelial cells [20]. BnSP-6, a Lys-49 PLA_2_ from *Bothrops pauloensis* venom, caused dose-dependent inhibition of adhesion, migration, stimulated the autophagy process, and induced both early and late apoptosis, which was confirmed by the modulation of different genes related to the apoptosis pathway [17]. BnSP-6 also induced genotoxic effects and DNA damage of triple-negative breast cancer cells [18].

The literature includes several findings that have demonstrated the action of various svPLA_2_ specifically against breast cancer cells [19,21,22,23,24]. In this regard, a pioneer study by Azevedo et al. in 2019 showed the antitumor and antimetastatic effects of PLA_2_ BthTx-II an Asp49-PLA_2_ isolated from *Bothrops jararacussu* venom [17,21]. Azevedo and colleagues (2019) reported that this protein was able to induce cell death, and inhibited cell proliferation, adhesion, migration, invasion, and growth of 3D structures (mammospheres); in addition, BthTx-II decreased the expression of important genes and proteins involved in the metastatic process, and inhibited the epithelial–mesenchymal transition (EMT) of this cell by increasing E-cadherin (CDH-1) and decreasing TWIST1, CTNNB1, vimentin and cytokeratin-5 expression. Finally, the authors demonstrated that triple-negative breast cancer cells after BthTX-II treatment assumed an epithelial phenotype, thus decreasing its aggressiveness [17].

BthTx-II is an important Asp49-PLA_2_ that has been well structurally and functionally characterized [15,17,22,23,25]. It possesses 122 amino acid residues, a molecular mass of 13,976 Da, and an isoelectric point of 8.2, with low phospholipase activity when compared to other Asp49-PLA_2_ basic and acidic PLA_2_ [15,22,23]. The three-dimensional structure of BthTx-II reveals that it is not able to bind calcium ions, because Ca^2+^ binding loop is distorted, and that it presents changes in the C-terminal region, with a configuration close to that of tyrosine (Tyr28) in the main chain [25], which could be responsible for its low catalytic activity when compared with other PLA_2_s [25]. This protein shows myotoxic, edematogenic and hemolytic effects and low phospholipase activity. Moreover, BthTx-II also induces platelet aggregation and secretion through multiple signal transduction pathways [24]. These effects are probably mediated by its low enzymatic activity or by the interaction with integrins and cell receptors, as described for some non-enzymatic Lys49 PLA_2_ from snake venoms [26,27,28,29].

Thus, in the present study, we assessed, for the first time, the antiangiogenic effect of Asp-49 PLA_2_ BthTx-II on the invasion, migration, and proliferation of HUVEC, as well as its capacity to inhibit the sprouting angiogenic process through an ex vivo aortic ring assay. Furthermore, in vivo tumor suppression and antiangiogenic activities were analyzed using MDA-MB-231 cells with Matrigel injected into the chorioallantoic membrane of chicken embryo (CAM). Given our findings, we highlighted a new possible role for BthTx-II as a potential prototype for the development of new tools and antiangiogenic drugs in cancer therapy.

## 2. Materials and Methods

### 2.1. Animals

The management, use, and euthanasia protocols of the animals were approved by the Animal Research Ethics Committee of the Federal University of Uberlândia, Brazil (CEUA/UFU 130/15), and for chicken embryos it was supported by final analysis number A011/20; every effort was made to minimize suffering.

### 2.2. Venom and Purification of Asp-49 PLA_2_ BthTx-II

*Bothrops jararacussu* crude venom was collected from snakes kept at the the Animal Toxin Extraction Center Ltd.—(CETA serpentarium), Morungaba, Sao Paulo, Brazil. This serpentarium provided proof of IBAMA registration and use of renewable natural resources (n° 2087163). BthTx-II was purified from *Bothrops jararacussu* venom as previously described by Azevedo (2019). The samples obtained were stored at −20 °C.

### 2.3. Cell Culture

The human breast cell lines MDA-MB-231 (triple-negative breast cancer subtype) and HUVEC (Human Umbilical Vein Endothelial Cells) were obtained from the American Type Culture Collection (ATCC^®^ HTB-26) (Manassas, VA, USA) and the American Type Culture Collection (ATCC^®^ CRL-1730), respectively, and maintained at 37 °C in a humidified incubator containing 5% CO_2_. MDA-MB-231 cells were grown in IMDM medium (Sigma, Brazil) containing 10% heat-inactivated fetal bovine serum (FBS) (Cultilab, Brazil). HUVEC were cultivated in RPMI 1640 medium supplemented with 10% heat-inactivated fetal bovine serum, 2 mM L-glutamine, 2 mM sodium pyruvate, 1 mM non-essential amino acids. The cell cultures were incubated at 37 °C in a humidified atmosphere of 95% air and 5% CO_2_ and all the cell lines used were kept free of mycoplasma and confirmed by PCR.

### 2.4. Cell Viability by MTT Assay

Briefly, HUVEC (3 × 10^4^ cells/well) were seeded into 96-well microplates and incubated for 24 h at 37 °C in 5% CO_2_. After adhesion, cells were treated with different concentrations of BthTx-II (ranging from 0.78 µg/mL to 50 µg/mL) in the presence or absence of VEGF (10 ng/mL) for 24 h. Control group cells were incubated in absence of BthTx-II or VEGF. Subsequently, cells were incubated with MTT solution (5 mg/mL, 20 μL/well) (Sigma, Brazil) for 3 h, and then, formazan crystals were dissolved by 100 μL of a solution containing 10% SDS and 0.01 M HCl in phosphate buffered saline (PBS) for 18 h. The optical density was determined at 570 nm in a plate reader (Multiskan GO Thermo Scientific, Waltham, MA, USA).

### 2.5. Proliferation Assay

HUVEC (2 × 10^4^ cells/per well) were seeded in a 24-well plate for four days at 37 °C in 5% CO_2_. Cell populations were normalized in serum-free medium for 24 h, after this step, the medium was aspirated and replaced by a medium containing BthTx-II (1, 10, 25 and 50 μg/mL). For positive control cells, RPMI medium with 10% FBS was used, while for negative control cells, IMDM medium was without FBS supplementation. All medium and treatment conditions were replaced every 24 h. After 72 h, 30 μL of MTT (5 mg/mL) (Sigma-Aldrich M2128, St. Louis, MO, USA) was added to each well and the plates were incubated for 3 h at 37 °C in 5% CO_2_. Formazan crystals were dissolved in 150 µL of DMSO for 15 min under gentle agitation. Absorbance was read at 570 nm using a microplate reader (Molecular Devices, Menlo Park, CA, USA). Blank spaces must produce values of 0 ± 0.1 O.D. units. The mean values of the triplicate readings were determined, and the mean blank value was subtracted. The data were expressed as O.D. values.

### 2.6. Cell Adhesion Assay

To verify the inhibition of cell adhesion, HUVEC (3 × 10^4^ cells/well) were pre-incubated with different concentrations of BthTx-II (ranging from 0.78 µg/mL to 50 µg/mL) for 40 min at 37 °C in 5% CO_2_. Control group cells were incubated with medium supplemented with 10% FBS. Subsequently, cells were seeded into 96-well microplates and incubated for 2 h at 37 °C in 5% CO_2_. After this procedure, the unattached cells were removed by gently washes with PBS. The adherent cells were measured by MTT assay, and the percentage of inhibition of cell adhesion was determined by software GraphPad Prism, version 9.0 (GraphPad Program Inc., San Diego, CA, USA).

### 2.7. Cell Cycle Analysis

Cell cycle analysis by DNA content was performed. Briefly, HUVEC were seeded at 2 × 10^5^ cells/well in 24-well microplates. After 24 h, cells were incubated with BthTx-II (10 and 50 μg/mL) in the presence or absence of VEGF (10 ng/mL) for 24 h at 37 °C in 5% CO_2_. As control group, HUVEC were treated with culture medium only. Then, cells were harvested, washed with PBS, and fixed in cold 70% (*v*/*v*) ethanol overnight at 4 °C. After this procedure, cells were washed with PBS and treated with RNase A (100 μg/mL) (Corning, São Paulo, Brazil) to ensure that only DNA was stained. Cells were stained with propidium iodide (PI) (10 μg/mL) (Sigma, São Paulo, Brazil) for 45 min at 37 °C. Immediately after incubation, the cell cycle was determined by the flow cytometer C6-ACCURI (BD Bioscience, WA, USA), and the data obtained were analyzed using FlowJo™ v10 Software—BD Biosciences, OR, USA.

### 2.8. Cell Migration Assay

Wound-healing was performed to assess the effect of BthTx-II on cell migration. In brief, 1 × 10^5^ cells/well were plated in 24-well microplates and grown to confluence. After 24 h of serum starvation, a rectangular lesion was made in the monolayer using a 200 μL pipette. Subsequently, cells were treated with BthTx-II (1, 10, 25 and 50 μg/mlL) diluted in supplemented culture medium containing VEGF (10 ng/mL). As a control group, cells were treated with culture medium supplemented with 10% FBS (300 μL) and medium with VEGF (control+ VEGF) for 24 h. To evaluate the wound-healing process, a microscopy (Nikon Eclipse TS100) was used to photograph the monolayer immediately after scratching (*t* = 0 h) and upon 24 h of treatment (*t* = 24 h). The cell migration on the created scratching edge was measured by Image J software and the percentage of wound closure was obtained by the following equation: Scratch Wound coverage = [(*A*_*t*__=0h_ − *A*_*t*__=24h_)/*A*_*t*__=0h_] × 100.

### 2.9. In Vitro Angiogenesis (Tubulogenesis Assay)

The vessel formation was evaluated in HUVEC by the Matrigel tube formation assay. The HUVEC (5 × 10^5^ cells/well) were pre-incubated with BthTx-II (10 and 50 µg/mL) or medium (control group) for 30 min at 37 °C in RPMI medium supplemented with basic fibroblast growth factor (bFGF) at 10 ng/mL. Next, the cells were seeded on a cell culture chamber coverslip (BD Bioscience, OR, USA) coated with 50 µL of Matrigel 5.25 mg/mL (Corning^®^ Matrigel^®^ Matrix, OR, USA) and maintained at 37 °C in a humidified incubator containing 5% CO_2_. After 18 h, cells were photographed in an inverted optical microscope (Nikon Eclipse TS100) and the vessels enumerated [30].

### 2.10. Dosing of VEGF in the Supernatant of HUVEC

The VEGF levels were quantified in the supernatant of the HUVEC obtained after in vitro vessel formation assay. This dosage was performed using a commercial CBA Kit for cellular proteins (BD Biosciences, OR, USA), following the manufacturer’s recommendations. The VEGF levels were calculated in comparison to a standard curve produced with recombinant VEGF (2500, 1250, 625, 312.5, 156, 80, 40, 20 and 10 pg/mL). The samples were acquired by flow cytometer BD Accuri C6 (Biosciences, WA, USA) and analyzed with the software FlowJo™ v10 Software—BD Biosciences, OR, USA.

### 2.11. Ex Vivo Angiogenesis Assay (Mouse Aortic Ring Assay)

This assay was performed according to Baker et al., 2011 [31] with modifications. Aortic fragments (1 mm–1.5 mm) were removed from male BALB/c mice (three animals per experiment, with 6 weeks old, according to statement approved by the Committee for the Ethical Use of Animals (CEUA) of the Federal University of Uberlandia (UFU), (CEUA, protocol number 130/15), and rinsed in ice-cold PBS supplemented with 1% penicillin-streptomycin. The fragments were placed on top of Matrigel (5.25 mg/mL, Corning^®^ Matrigel^®^ Matrix, OR, USA) coated on 24-well plates and incubated in RPMI medium supplemented with bFGF (10 ng/mL) for 24 h before being treated with BthTx-II (10 and 50 μg/mL) or medium (control group). These fragments were treated over 9 days, at 1-day intervals. Subsequently, the aortic rings were photographed using an inverted optical microscope (Nikon Eclipse TS100) [32] and the sprouting length was expressed in pixels for quantitative analysis in ImageJ software, as previously described by [33].

### 2.12. Characterization of Co-Culture HUVEC and MDA-MB-231 Cells and Angiogenic Sprouting

The 3D co-culture assay was performed according to Swaminathan (2017) with some modifications. Previously, HUVEC were stained in red (BD Pharmingen™ MitoStatus Red, San Diego, CA, USA) and MDA-MB-231 cells stained with carboxyfluorescein succinimidyl ester (CFSE) to better visualize the interaction between the cells. The plates were previously coated with 200 µL of Matrigel (Corning^®^ Matrigel ™, OR, USA), solidified for 40 min; HUVEC and MDA-MB-231 cells were added to the wells individually and incubated in 300 µL of fresh medium enriched with growth factors for up to 48 h until the formation of 3D structures. After that time, newly formed MDA-MB-231 cell spheroids (according to the 3D methodology) were transferred to the newly formed Matrigel™ endothelial cell vessel network. This network of endothelial vessels co-cultured with 3D mammary spheroids allows the interaction of an important building block for studying physiologically relevant cell–cell interactions. For this assay, we used a 1:1 ratio between tumor cells and endothelial cells (exact numbers: 1200 tumor cells, 1200 endothelial cells) in the RPMI medium to assess the interaction between tumor cells and endothelium [34].

### 2.13. Co-Culture Invasion Assay

Co-culture model was performed on 4.0 µm PET membrane to separate HUVEC and MDA-MB-231 cells into different compartments of the same well. One hour prior to co-culture, HUVEC (5 × 10^4^ cells) were seeded in 500 μL of RPMI medium in 24-well plates with or without a Matrigel coating (BD Biosciences, OR, USA), and then the same number of MDA-MB-231 cells were seeded into the upper chamber of the transwell membrane, and then placed directly on top of the 24-well plate containing the HUVEC. The tests were executed in the presence or absence of growth factors (EGF and bFGF), and then treatment with BthTx-II (10 and 50 μg/mL) was performed or medium was added to the upper wells to allow invasion to be verified after 24 h.

### 2.14. Chick Chorioallantoic Membrane Angiogenesis Assay

For the evaluation of angiogenesis in the chicken chorioallantoic membrane (CAM), we used a process previously described in [19], with some modifications. Chick embryos were opened from 3-day-old eggs, and 1 mL of albumen was removed to detach the chorioallantoic membrane. After 5 days at 37 °C, a suspension was prepared containing 1 × 10^6^ cells MDA-MB-231 in Matrigel (20 µL) and VEGF 200 ng/mL. After 3 days, BthTx-II (10 and 50 µg/mL) was applied on CAMs, while the treatment with PBS p.H 7.4 was considered positive control; after 4 days, the angiogenic and antitumor response were evaluated and photographed. After the experimental period, the tumor was retrieved from CAM for evaluation of size and weight.

### 2.15. Statistical Analysis

Results are shown as means ± standard errors of the mean (SEM) of at least 3 independent experiments. The software GraphPad Prism, version 9.0 (GraphPad Program Inc., San Diego, CA, USA) was used to determine differences between mean values with ANOVA or *t*-test. *p* values of less than 0.05 were considered statistically significant.

## 3. Results

### 3.1. BthTx-II Induces Cytotoxicity and Decreases the Proliferation of HUVEC

**Figure 1:**
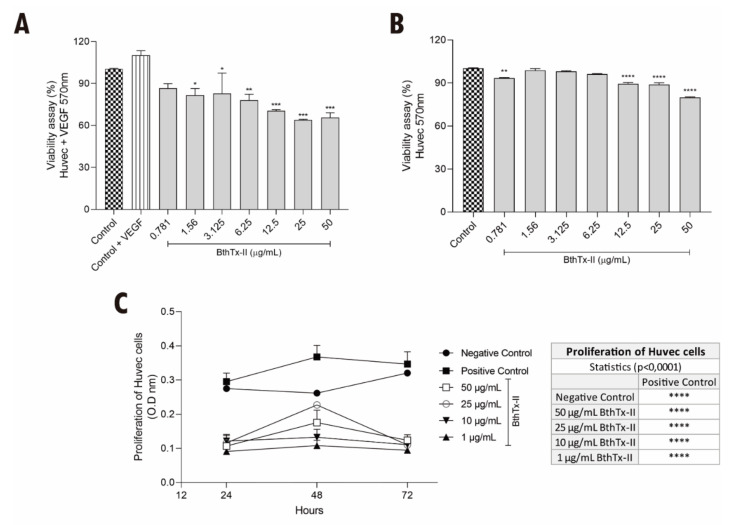
BthTx-II induces cytotoxicity and inhibits proliferation in HUVEC. (**A**) Interference of BthTx-II in the viability of HUVEC in the presence of VEGF with different concentrations (0.781, 1.56, 3.125, 6.25, 12.5, 25, and 50 μg/mL) by MTT assay. (**B**) Interference of BthTx-II in the viability of HUVEC in the absence VEGF with different concentrations (0.781, 1.56, 3.125, 6.25, 12.5, 25, and 50 μg/mL) by MTT assay. (**C**) Effects of BthTx-II (1, 10, 25, and 50 μg/mL) on proliferation of HUVEC for 24, 48 and 72 h of treatment, and the table on the side shows the statistical values for each treatment in relation to the positive control. Values represent the mean ± SD (*n* = 3) of three independent experiments, analyzed by two-way ANOVA and Bonferroni post hoc test. Statistical difference (* *p* < 0.1, ** *p* < 0.01, *** *p* < 0.001 and **** *p* < 0.0001) is with reference to untreated cell control.

To evaluate the effect of BthTx-II on the viability of HUVEC stimulated or not with VEGF, the MTT assay was performed. As shown in Figure 1A, the simultaneous treatment with VEGF and BthTx-II caused cytotoxicity of around 40% at the highest concentrations of the toxin (i.e., 12.5, 25 and 50 µg/mL) compared to HUVEC without VEGF treatment. Similarly, BthTx-II (12.5, 25 and 50 µg/mL) also reduced the viability of HUVEC in the absence of VEGF (Figure 1B). In addition, BthTx-II was able to significantly abolish the proliferation of HUVEC at all concentrations tested in 24, 48 and 72 h (Figure 1C).

### 3.2. BthTx-II Promotes G0-G1 Phase Cell Cycle Arrest in HUVEC

**Figure 2:**
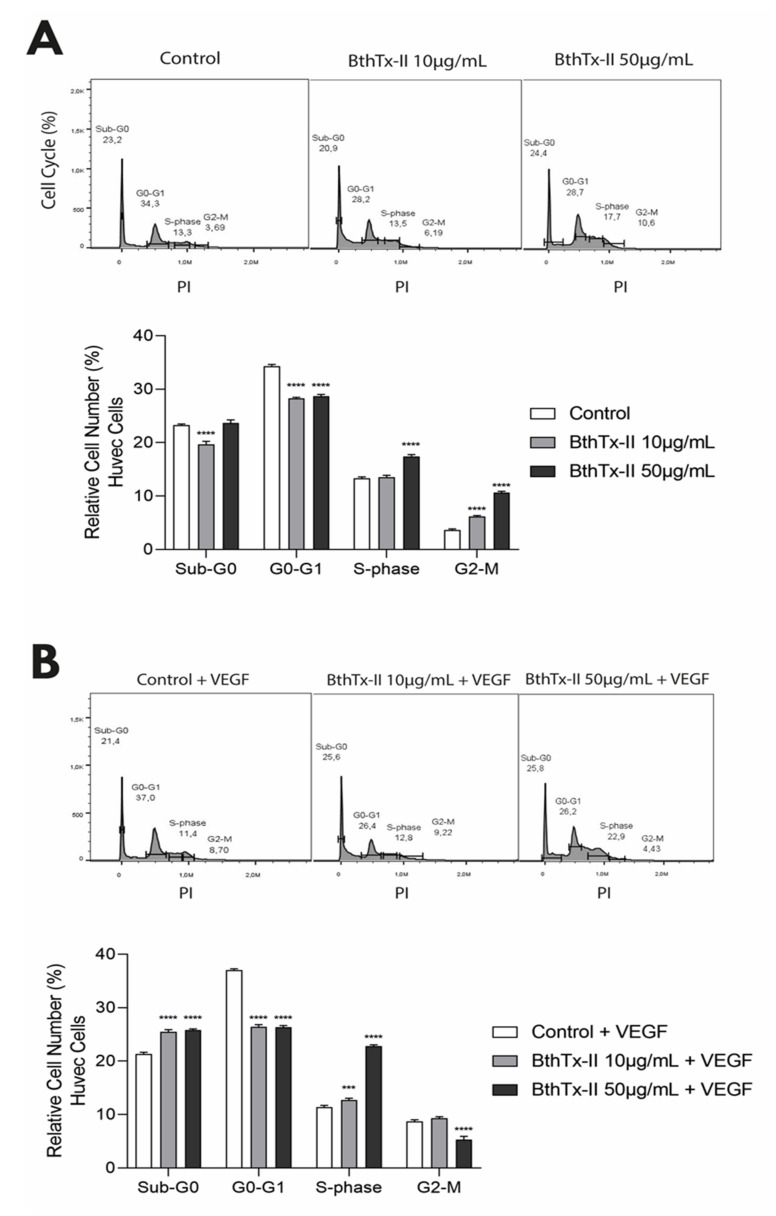
BthTx-II differently modulates cell cycle phases of HUVEC in the presence or absence of VEGF. Cell cycle histogram profile of HUVEC, and representative graph after treatment with different concentrations of BthTx-II (10 and 50 μg/mL) in the (**A**) absence or (**B**) presence of VEGF for 24 h. *** *p* < 0.001, and **** *p* < 0.0001 when compared to the respective control group. Data are expressed as mean ± standard error of experiments performed at least three times independently in triplicates.

The effect of BthTx-II on cell cycle progression was evaluated in HUVEC treated with BthTx-II for 24 h and labeled with propidium iodide (PI). Figure 2A shows the histogram profile of the cell cycle of HUVEC after treatment with BthTx-II in the absence of VEGF for 24 h. After treatment with BthTx-II (10 and 50 μg/mL), the cell population in G0/G1 decreased to 28.2% and 28.7%, respectively, in comparison with 34.3% in control cells (untreated group) (Figure 2A). In addition, a slight decrease (20.9%) in cell population in sub-G0 was observed after treatment with BthTx (10 μg/mL) when compared to 23.2% control (Figure 2A) Moreover, both concentrations of BthTx-II promoted an arrest in G2-M phase (Figure 2A). When HUVEC were stimulated with VEGF, BthTx-II (10 and 50 μg/mL) decreased the cell number in G0-G1 phase to 26.4% and 26.2%, respectively, when compared to the 37% of the control (Figure 2B). In addition, both concentrations of BthTx-II caused an arrest in sub-G0 phase related to the control (Figure 2B).

### 3.3. BthTx-II Interferes with the Adhesion and Migration of HUVEC

**Figure 3:**
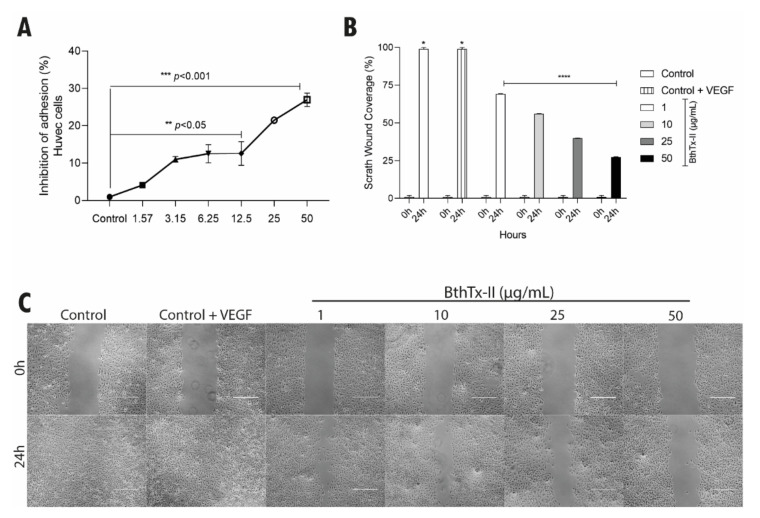
BthTx-II inhibited adhesion and migration of HUVEC. (**A**) Interference of BthTx-II in the adhesion of HUVEC pre-incubated with BthTx-II (1.57, 3.15, 6.25, 12.5, 25, and 50 μg/mL) for 30 min. (**B**) Percentage of inhibition of migration by wound-healing assay when HUVEC were treated with BthTx-II (1, 10, 25, and 50 μg/mL) with VEGF. (**C**) Photograph of HUVEC wound-healing assay. The cell monolayer was grown to confluence and at experimental time t = 0 a slit was made. Each well was photographed at time t = 0 and t = 24 h after treatment with BthTx-II. Values represent the mean ± SD (*n* = 3) of three independent experiments, analyzed by two-way ANOVA and Bonferroni post hoc test, as well as statistical difference (* *p* < 0.1, ** *p* < 0.05, *** *p* < 0.001 and **** *p* < 0.0001) in comparison to untreated cell control.

Different BthTx-II concentrations (1.57 to 50 μg/mL) inhibited HUVEC adhesion, with the highest concentration (50 µg/mL) inhibiting adhesion by approximately 28% with respect to the control (Figure 3A). The wound-healing assay demonstrated that BthTx-II (1, 10, 25 and 50 μg/mL) was able to significantly reduce HUVEC migration after 24 h of treatment when compared to the control groups (Figure 3B,C).

### 3.4. BthTx-II Inhibits Vessel Formation and Decreases the Production of Endothelial Growth Factor (VEGF) (In Vitro Model)

**Figure 4:**
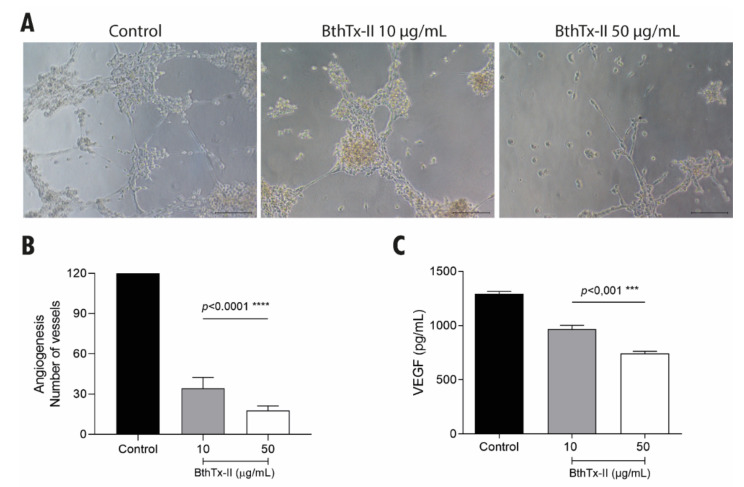
Inhibition of vessel formation in vitro by BthTx-II. (**A**) Representative photomicrograph of the Matrigel tube formation assay of HUVEC induced with bFGF in the absence or presence of BthTx-II (10 and 50 μg/mL). (**B**) Graphic representation of the quantification of the number of vessels formed by HUVEC cells after 18 h. (**C**) Quantification of the VEGF levels released in the supernatant of HUVEC. All independent experiments were performed in triplicates, and values represent the mean ± SD (*n* = 3). Differences compared between treatments and control groups were analyzed by one-way ANOVA. Statistically significant values are represented by *** *p* < 0.001 and **** *p* < 0.0001. Scale bar, 200 µm.

BthTx-II (10 and 50 μg/mL) treatment significantly reduced vessel formation in Matrigel™, even in the presence of basic fibroblast growth factor (bFGF) (Figure 4A). In this assay, an average of 120 vessels was quantified in the control group, 30 vessels in the group treated with BthTx-II (10 μg/mL), and 10 vessels after treatment with 50 μg/mL (Figure 4B). The VEGF levels were quantified in the supernatant of HUVEC after treatment with BthTx-II (10 and 50 μg/mL). This toxin was able to reduce VEGF levels to 960 and 740 pg/mL, respectively, when compared to control (1250 pg/mL) (Figure 4C).

### 3.5. BthTx-II Inhibits the Formation of Vessels through the Aortic Ring of Mice

**Figure 5:**
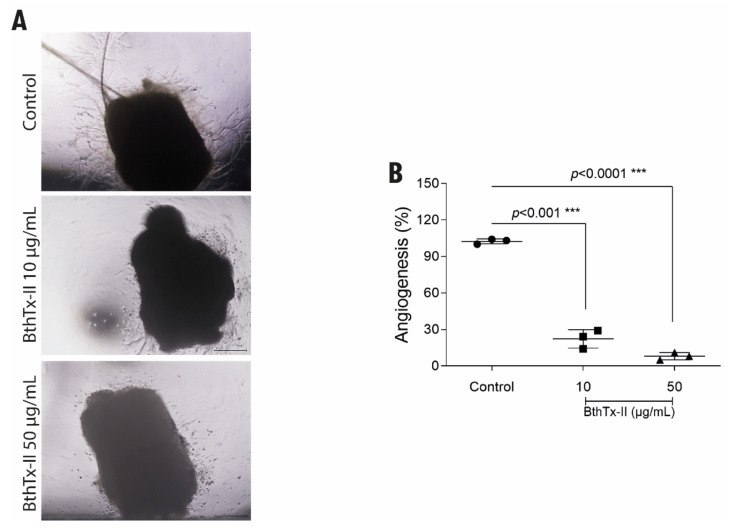
BthTx-II promotes a decrease in vessel formation in an ex vivo model (aortic ring assay). (**A**) Representative images of microvessels from aortic fragment removed from mice in an ex vivo model after 7 days of treatment with BthTx-II (10 and 50 μg/mL). (**B**) Graphic representation of the quantification of the number of microvessels formed by the aortic ring sprouting formed in the control compared to the treatments. Differences compared between treatments and control were analyzed by one-way ANOVA. Statistically significant values are represented by *** *p* < 0.001. Scale bar, 200 µm.

To assess the ex vivo antiangiogenic effect of BthTx-II, an aortic ring assay was performed. Both concentrations of BthTx-II reduced the number of cellular microvessels formed from fragments of aortic vessels (Figure 5A). The groups treated with BthTx-II (10 and 50 μg/mL) over 7 days at 2-day intervals and compared to the untreated control revealed a 70% reduction in the lowest dose and 90% in the highest dose (Figure 5B).

### 3.6. BthTx-II Inhibits Transwell/Matrigel Invasion of MDA-MB-231 Cells: HUVEC and Tumor Angiogenesis Assessed by Co-Culture Assay

**Figure 6:**
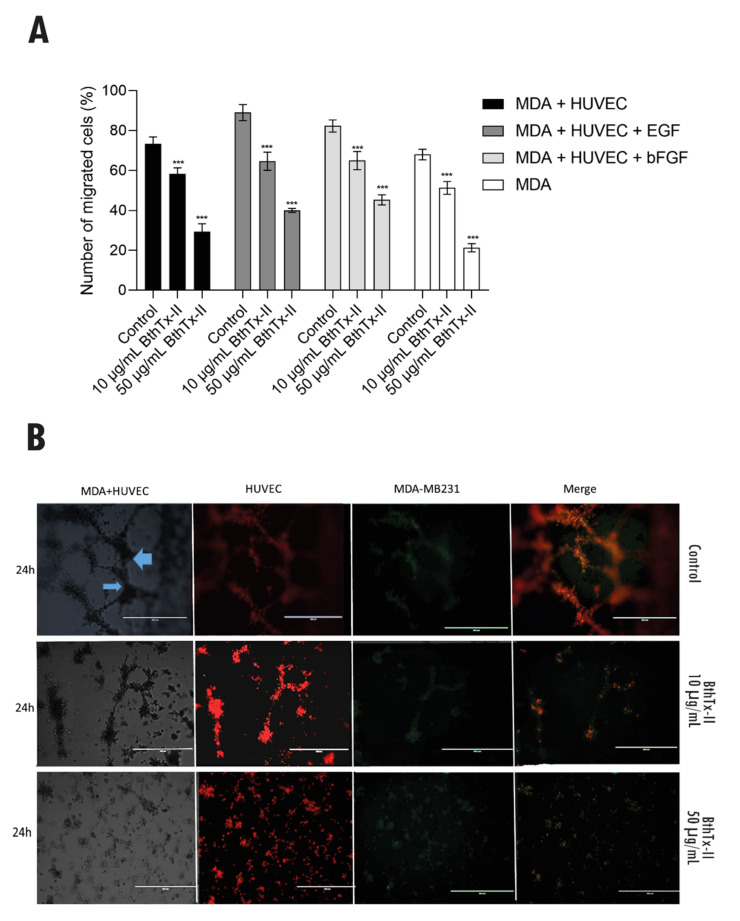
Inhibition of co-culture invasion of treated cells with BthTx-II. (**A**) Inhibition of cell invasion in MDA-MB-231 co-culture in Matrigel-HUVEC in transwell and action of BthTx-II (10 and 50 µg/mL) in the presence or not of stimuli with EGF and bFGF. (**B**) Tumor angiogenesis, HUVEC/MDA-MB-231 co-culture. Control MDA-MB-231 and HUVEC, in co-culture, representing a tumor angiogenesis model. Blue arrows represent the meeting of TNBC cell spheroids with newly formed endothelial vessels. Treatment with BthTx-II (10 µg/mL) in cell co-culture, with inhibition of cell migration and proliferation. Treatment with BthTx-II (50 µg/mL), with full effect on cell migration and proliferation inhibition, after 24 h of treatment and at both concentrations. All experiments were performed in independent triplicates, and values represent mean ± SD (*n* = 3). Differences compared between treatments and cell control without co-culture were analyzed by two-way ANOVA. Statistically significant values were represented by *** *p* < 0.001.

Figure 6A shows the inhibition of invasion of MDA-MB-231 cells co-cultured with HUVECs in a transwell/Matrigel assay when treated with BthTx-II in the presence or absence of different stimuli (EGF and bFGF). MDA-MB-231 cells showed an invasive capacity in Matrigel of 73% and 63% when cultured in the presence or absence of HUVEC cells, respectively. MDA-MB-231 cells were also stimulated with EGF and bFGF and were able to invade in 89% and 82.3% of cases after the described stimuli, respectively. BthTx-II (10 µg/mL) inhibited the invasion of MDA-MB-231 cells by 40% when subjected to the co-culture system with HUVEC. Even in the presence of growth factors, the toxin inhibited EGF-stimulated invasion by 42% and 45% by bFGF, when in a co-culture system (MDA-MB-231 + HUVEC). Furthermore, when MDA-MB-231 cells, in the absence or presence of growth factors, as well as in the co-culture system, were treated with BthTx-II (50 µg/mL), they showed a 70% to 80% inhibition of invasion.

To assess the interaction of newly formed tubules of endothelial cells with breast cancer epithelial cell spheroids, a 3D cell co-culture was performed. Figure 6B shows the control groups (MDA-MB-231 + HUVEC), where the MDA-MB-231 cells spheroids are in contact with the endothelial vessels, represented by the blue arrows. In subsequent images, HUVEC (labeled in red), and MDA-MB-231 cells (labeled in green) allow their identification in TNBC cell clusters for a period of 24 h in the presence or absence of BthTx-II (10 and 50 µg/mL). Treatment with BthTx-II (10 µg/mL) exhibits an inhibitory effect on cell aggregation, especially on endothelial cells. In addition, treatment with BthTx-II (50 µg/mL) exhibits an effect on the total inhibition of cell co-culture migration and proliferation for a period of 24 h, compared to cell control in Matrigel. It is also important to note that the co-culture of HUVECs and MDA-MB-231 cells was able to induce changes in their morphology due to their interaction.

### 3.7. BthTx-II Inhibits Tumor Angiogenesis In Vivo through the Chick Chorioallantoic Membrane (CAM)

**Figure 7:**
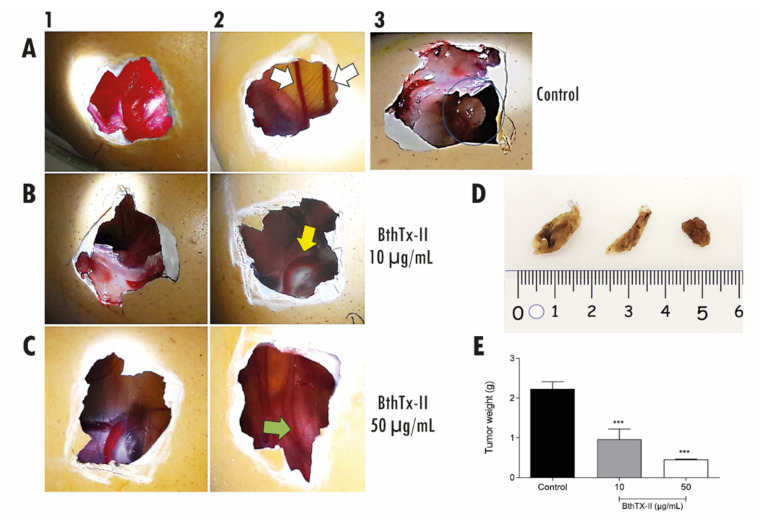
In vivo tumor angiogenesis by CAM assay. (**A1**) Control and tumor growth. (**A2**) Formation of new vessels. (**A3**) Tumor developed. (**B1**) Start of treatment with BthTX-II (10 µg/mL). (**B2**) After 4 days of treatment with BthTX-II (10 µg/mL). (**C1**) Start of treatment with BthTX-II (50 µg/mL). (**C2**) After 4 days treatment with BthTX-II 50µg/mL. (**D**) Tumor reduction photo after treatment with BthTx-II represented and ordering by control, 10 and 50 µg/mL. (**E**) Weight of tumors (g). Statistically significant values were represented by *** *p* < 0.0001.

In this assay, 25 matrices of fertilized hen eggs were used with the developing embryo incubated at 9 days of life with TNBC cells in Matrigel. During incubation, the viability of the chicken embryo was evaluated daily, as a prerequisite for carrying out the experiment and verifying its viability. Tumor growth was observed in 18 eggs (72%). After 3 days of inoculation, there was new vessel formation (thin white arrow) from the pre-existing ones (Figure 7A2), and the tumor had already been installed at an advanced stage (Figure 7A3). Treatment with BthTx-II (10 and 50 μg/mL) was performed every day from the 4th day of tumor inoculation, with vessel formation and tumor growth being observed daily. Figure 7B2 demonstrates the reduction in tumor mass after treatment with BthTx-II (10 µg/mL). There was a reduction in vessel caliber (Figure 7C), tumor size (Figure 7D), and tumor weight (Figure 7E) after treatment. As shown in Figure 7E, tumor weight was reduced by about 52% and 82% compared to the control after treatment with BthTx-II at concentration of 10 μg/mL and 50 μg/mL, respectively.

## 4. Discussion

Snake venom phospholipases A_2_ (svPLA_2_) have been widely explored for their antitumor potential [20,21,22]. These proteins have high potential in cancer therapy, since they can recognize and act on different death pathways, inhibiting processes such as cancer invasion, proliferation, metastasis, and angiogenesis [19,21,31,32].

Azevedo et al. (2019) [17] previously demonstrated that BthTx-II was able to inhibit tumor growth, evoking mechanisms of death, autophagy, and regulating the expression of pro-apoptotic genes. Furthermore, it has been reported that BthTx-II inhibits the migration, adhesion, and invasion of TBNC cells in the integrin-mediated cell cycle and interferes with the epithelial–mesenchymal transition process of highly metastatic breast tumor cells (MDA-MB-231 cells) by reducing the expression of important genes and proteins related to the metastatic process, such as CK-5, Vimentin, CTNNB1, and TWIST1, and by increasing E-cadherin (CDH-1) [17].

In this study, we assessed the antiangiogenic and antimetastatic effects of BthTx-II using in vitro, ex vivo, and in vivo assays. The antiangiogenic activity of BthTx-II was initially verified through the cell viability and proliferation of monolayer HUVEC subjected or not to stimuli with VEGF, which is a bioactive protein capable of exerting angiogenic, mitogenic, and vascular permeability-increasing activities [35]. Interestingly, BthTx-II (10 and 50 µg/mL) promoted a reduction in the viability of HUVEC, even after stimulation with VEGF, a fact that occurred less significantly with the Asp-49-PLA_2_-Pllans-II from *Porthidium lansbergii lansbergii* snake venom [36].

In order to obtain insights into the antiangiogenic effect of BthTx-II, we assessed the cell cycle progression of HUVEC. Our data demonstrated that the presence of VEGF could differently modulate the cycle phases of BthTx-II-treated HUVEC. We observed that, in the absence of VEGF, BthTx-II reduced G0-G1 phase, but promoted G2-M arrest. In agreement with this, it was demonstrated that BnSP-6, a Lys-49-PLA_2_ homologue from *B. pauloensis* snake venom, promoted reduction in the number of MDA-MB-231 cells in the G0-G1 phase, and augmentation in the G2/M phase, by modulation of important genes related to cell cycle progression [18].

Conversely, we observed that simultaneous treatment with VEGF and BthTx-II also decreased the number of cells in G0-G1 phase, but arrested the cells in sub-G0. Corroborating our data, Polloni et al. 2021 reported that BnSP-7, a Lys-49-PLA_2_ from *B. pauloensis* snake venom, promoted a reduction in the number of HUVEC in G0-G1 phase, and arrested the cells in sub-G0. Similarly, it was demonstrated that crotoxin and cytotoxin-III, other snake toxins, also acted by reducing the cell number in G0-G1 phase [37,38]. The accumulation of cells in sub-G0 and their reduction in G0-G1 phase has been widely associated with DNA fragmentation, which can activate cell cycle checkpoints, preventing progression to the next phase of the cell cycle until the damage is repaired; however, when the DNA damage is irreparable, programmed cell death may be activated [39]. Despite the differences observed, with or without VEGF stimulation, BthTX-II was able to cause a disturbance in cell cycle progression, which was corroborated by the proliferation assay, where the toxin inhibited HUVEC growth. Therefore, the ability of BthTx-II to promote disturbance in the cell cycle process suggests that this toxin may constitute a highly attractive therapeutic strategy.

Taken together, our findings show that BthTx-II can assume different roles in the presence or absence of VEGF, which can be explained, at least in part, by the permeability action being mediated by VEGF. It has been reported that VEGF is capable of inducing extracellular proteolytic effects that can increase cell permeability [40]. Moreover, it has been reported that BthTx-II is able to augment the membrane permeability of breast cancer cells [17,41]. Thus, we suggest that the increased membrane permeability caused by VEGF could potentialize BthTx-II activity in the promotion of cell permeability, corroborated by the higher rates of cytotoxicity, and the different profile of cell cycle observed in HUVEC. However, the underlying mechanisms by which BthTx-II interacts directly or indirectly with VEGF need to be thoroughly evaluated in future works.

BthTx-II reduced adhesion and migration in HUVEC, as well as demonstrating the ability to inhibit the formation of new vessels in Matrigel (in vitro angiogenesis) in the presence of bFGF. Similar results were also observed for a recombinant snake venom, cystatin, which shows an antiangiogenic effect in vitro and in vivo through the inhibition of VEGF and bFGF production [42]. Bothropoidin, a disintegrin-like metalloproteinase isolated from *B. pauloensis* snake venom, and BnSP-7 were able to decrease human endothelial cell viability and adhesion to Matrigel, as well as to inhibit in vitro angiogenesis in bFGF-stimulated Matrigel [14,43].

svPLA_2_s can act on specific target sites, including cell membranes and/or cell receptors of determined tissues [23,44]. The pharmacological effects triggered by svPLA_2_s can occur through their interaction with phospholipids and/or proteins, in which the most described svPLA_2_s receptors in cell membranes are integrins and other receptors, such as vascular endothelial growth factor receptor (VEGFR)-2 [26,45,46], M-type receptors [47] and nucleolin [48]. Thus, we hypothesized that the antiangiogenic effects of BthTx-II occur due to their ability to bind to specific sites on HUVEC, resulting in a downstream activation of distinct intracellular pathways. Corroborating this line of discussion, our data demonstrated a reduction in the VEGF levels present in the supernatant of BthTx-II-treated HUVEC, which could partially explain the inhibition of the formation of vessels. As shown in this study, antiangiogenic effects by others snake toxins have been described. MVL-PLA_2_, from *Macrovipera lebetina* snake venom, significantly inhibited angiogenesis in vitro and in vivo through actin cytoskeleton remodeling and by disturbing the distribution of α5β3 integrin, a critical regulator of angiogenesis and a major component of focal adhesions [41]. svPLA_2_-BnSP-7 also impaired the angiogenesis process via a negative modulation of the protein expression of VEGF [14].

It is worth mentioning that VEGF has an important pro-angiogenic activity, with mitogenic and anti-apoptotic effects on endothelial cells. It increases vascular permeability, and it is responsible for stimulating the proliferation and migration of endothelial cells during the process of tumor angiogenesis [49,50]. In this context, an intriguing question is raised: how does the Asp-49-PLA_2_ BthTx-II interfere with VEGF production? It was demonstrated that Lys-49-PLA_2_ isolated from *Agkistrodon piscivorus piscivorus* snake venom is able to bind to the extracellular domain of the kinase insert domain-containing receptor (KDR; also known as VEGF receptor-2) with high affinity via a C-terminal loop region, resulting in specific blockade of endothelial cell growth [45,46]. Thus, we suggested that BthTx-II can act through a direct interaction with this receptor or by other distinct target sites. Furthermore, subsequent mechanisms by which BthTx-II inhibits the angiogenesis process need to be thoroughly evaluated in future studies.

To corroborate and reinforce the findings regarding the inhibition of in vitro vessel formation induced by BthTx-II, in this study, we also evaluated the aortic behavior (ex vivo) in the formation of new vessels originating from a pre-existing vessel from budding endothelial cells. The intimal endothelium of the aorta has complete angioformative capacity and was a major contributor to angiogenic growth [19,27,29,48]. The results showed that after treatment with BthTx-II, at both concentrations tested, there was a reduction in the ability to form new vessels from pre-existing vessels when compared to the control without treatment, a fact that was evaluated for a period of 7 days. In agreement with our findings, this same effect has been reported for other snake venom toxins, such as CC5 and CC8 (two homologous disintegrins from *Cerastes cerastes* venom), and BnSP-7 [14].

An interconnected network mimicking a tumor microenvironment in vitro is extremely important for simulating angiogenesis and resembles conditions in vivo, in addition to highlighting that intercellular communication plays a key role in the progression and invasion of cancer, probably due to the vascularization of tumor tissue, and the modification of cell behavior, secreting a variety of growth factors, chemokines and proteases that play a direct role in resistance to targeted therapies and tumor recurrence in breast cancer [51,52]. Thus, we analyzed a 3D tumor angiogenesis co-culture model that allows for more targeted evaluation of antiangiogenic therapies. HUVEC cultured in co-culture with MDA-MB-231 triple-negative breast cells (TNBC) demonstrated greater invasiveness after co-culture. It is important to note that this co-culture, stimulated with bFGF and EGF growth factors, makes the TNBC cell more resistant and more invasive to the vascular endothelium [46,51,53].

The action of BthTx-II on cells (MDA-MB-231 and HUVEC) significantly inhibits the effects of invasion, representing more than 60% of this inhibition capacity. Studies obtained with breast cancer cells and HUVEC in co-culture endothelium–epithelium created from 3D structures were promisingly evaluated using stromal cell culture to study potential new antitumor compounds. A study with the BjussuLAAO-II from *Bothrops jararacussu* demonstrated a cytotoxic and genotoxic effect in a HepG2-HUVEC cell co-culture model [54]. These data suggest that factors released by tumor cells can positively regulate the expression of receptors on tumor-associated endothelial cells, and that the communication networks that are established between tumor cells and non-neoplastic cells in the microenvironment of primary tumors play a role crucial in the growth and development of metastases [52,55].

Chick embryo chorioallantoic membrane (CAM) assays have been widely studied in several types of cancers as an important target for suppressing their proliferation, survival, metastasis and angiogenesis in glioma tumors, osteosarcoma, and cervical carcinoma; all studies report the feasibility of using the chicken embryo as an alternative model system for analyzing various aspects of tumor biology [56,57,58,59,60].

Inhibition of BthTx-II-induced tumor growth and angiogenesis have also been analyzed in vivo using the CAM model [60]. After treatment with BthTx-II (10 and 50 µg/mL) for a period of 7 days, the tumor weight was reduced by 52% and 80%, respectively. It is important to report that at the end of the experiment, all embryos were alive, and that the treatment was not toxic to the embryo. It has been reported that CC-PLA_2_-1 and CC-PLA_2_-2 purified from *Ceraste ceraste* wax venom showed antiangiogenic activity in the CAM assay, decreasing vessel formation performed under the induction of angiogenesis by bFGF or VEGF [19].

To best of our knowledge, this is the first report of a PLA_2_ that has antitumor, antimetastatic and antiangiogenic effects to be evaluated in a CAM model using triple-negative breast cancer cells. We thus demonstrate that these combined effects in a single molecule directly impact tumor progression, highlighting BthTX-II in the scenario of the prototyping process of new antitumoral and anti-angiogenic agents.

## 5. Conclusions

In conclusion, the present work demonstrated, for the first time, the antimetastatic and antiangiogenic effects of PLA_2_-BthTX-II. BthTx-II was able to inhibit cell proliferation, adhesion, migration, and invasion. Furthermore, this toxin also inhibited the growth of new vessels in vitro and ex vivo; in addition, BthTX-II impaired the invasion and growth in co-culture of HUVEC and MDA-MB-231 cells. In the in vivo model in chicken embryos (CAM), the toxin reduced the tumor mass and the angiogenesis process in the membrane assay (CAM). Given the effects presented, we demonstrated the great potential of BthTx-II against breast cancer and tumor angiogenesis, showing that the results of this study suggest that BthTx-II can become a potential prototype for the development of tools for the study of antitumor therapies for triple-negative breast cancer.

## Data Availability

Data available in a publicly accessible repository that does not issue DOIs Publicly available datasets were analyzed in this study. This data can be found here: https://repositorio.ufu.br/?locale=pt_BR (accessed on 2 February 2021).
